# African Tickbite Fever in Travelers, Swaziland

**DOI:** 10.3201/eid1302.060808

**Published:** 2007-02

**Authors:** Paul M. Oostvogel, Gerard J. van Doornum, Russouw Ferreira, Jacqueline Vink, Florence Fenollar, Didier Raoult

**Affiliations:** * Medical Center Haaglanden, The Hague, the Netherlands; † Erasmus University Hospital, Rotterdam, the Netherlands; ‡ Skukuza, Republic of South Africa; § Université de la Mediterranée, Marseille, France

**Keywords:** Rickettsia africae, African tickbite fever, Swaziland, letter

**To the Editor:** African tickbite fever (ATBF), which is caused by *Rickettsia africae*, is well documented in travelers to southern Africa (*1*–*3*) and transmitted by ungulate ticks of the genus *Amblyomma*. Positive serologic results were reported in 9% of patients (*1*) and 11% of travelers (*4*) from southern Africa. We report an outbreak of ATBF with an attack rate of 100% among 12 Dutch travelers to Swaziland.

The 12 travelers (9 male and 3 female) visited Mkhaya Game Reserve in Swaziland in May 2003 for several days. Upon retuning to the Netherlands, they consulted our clinic for assessment for fever, malaise, and skin eruptions. Epidemiologic and clinical data were obtained after the patients provided informed consent. All symptomatic patients were treated before serum samples were collected.

Acute-phase and convalescence-phase serum samples were obtained from 8 patients at 3 and 9 weeks, respectively, after symptoms were reported. Only convalescent-phase serum samples were obtained from the other 4 patients. Serologic assays were conducted for screening and confirmation in Rotterdam, the Netherlands (Department of Virology, Erasmus University Hospital) and Marseille, France (Unité des Rickettsies, Faculté de Médecine, Université de la Mediterranée), respectively.

In Rotterdam, immunofluorescence assays for immunoglobulin G (IgG) and IgM against *R*. *conorii*, *R*. *typhi*, and *R*. *rickettsii* were performed with multiwell slides on which antigens were fixed (Panbio Inc., Columbia, MD, USA). Serum samples with fluorescent rickettsiae at dilutions >1:32 were considered positive.

In Marseille, a microimmunofluorescence assay for IgG and IgM against *R*. *africae*, other members of the spotted fever group, and *R*. *typhi* of the typhus biogroup was used. Western blotting for *R*. *africae* and *R*. *conorii* was performed with reactive serum samples and repeated after cross-adsorption that removed only antibodies to *R*. *conorii* (*5*). Serologic evidence for infection with *R*. *africae* was defined as 1) seroconversion; 2) IgG titers >64, IgM titers >32, or both, with IgG and IgM titers >2 dilutions higher than any of the other tested spotted fever group rickettsial antigens; 3) a Western blot profile that showed *R*. *africae*–specific antibodies; and 4) cross-adsorption assays that showed homologous antibodies against *R*. *africae* (*1*).

All 12 travelers had a diagnosis of ATBF. Epidemiologic, clinical, and serologic results are shown in the Table. Two patients had a history of a tickbite. Lymphadenopathy in the groin was the only clinical sign observed in 2 other patients. For all 10 patients with symptoms, the symptoms abated within a few days after treatment with doxycycline, 100 mg orally twice a day (5 patients) for 7 days, or ciprofloxacin, 500 mg orally twice a day (5 patients) for 7 days. No relapses or complications were noted 1 year later.

Assays in both locations showed serologic reactivity against *R*. *conorii* and *R*. *rickettsiae*. Specific antibodies against *R*. *africae* were detected by Western blot in 8 patients (Table). All 12 travelers were infected with *R*. *africae*. In 3 other patients, immunofluorescence assays demonstrated seroconversion for specific antibodies. One patient with no clinical symptoms had low IgG (32) and IgM (16) titers against rickettsiae by immunofluorescence and IgG by Western blot.

**Table:** Clinical and serologic characteristics of 12 travelers with African tickbite fever, Swaziland, 2003*

T	Sex/ age, y	Fever/headache/ myalgia/rash	Tickbite/ eschar site/ lymph. site	Sera	Rotterdam, the Netherlands		Marseille, France			
					*Rickettsia conorii* IgG/IgM†	*R. rickettsii* IgG/IgM†	*R. conorii* IgG/IgM†	*R. africae* IgG/IgM†	WB	WB ads.
1	F/47	Y/N/Y/Y	N/N/groin	A	0/32	32/32	0/0	0/0	NT	NT
				C	>128/>128	>128/32	128/0	128/0	+	Ra
2	M/14	Y/Y/N/N	Y/foot/groin	A	>64/>64	>64/>64	32/0	64/0	+	Ra
				C	>128/32	>128/16	256/0	256/0	NT	NT
3	M/13	N/N/N/N	N/N/N	A	0/0	32/0	0/16	0/16	+	NC
				C	0/0	0/0	0/16	0/16	NT	NT
4	M/10	N/N/N/N	N/N/groin	A	>64/>64	>64/>64	0/16	0/32	+	Ra
				C	>128/32	>128/16	0/64	0/64	NT	NT
5	M/50	Y/Y/N/Y	N/N/groin	A	0/0	0/0	0/0	0/0	NT	NT
				C	>128/>128	>128/32	64/8	64/8	+	NC
6	M/13	N/N/N/N	N/N/groin	A	0/0	0/0	0/0	0/0	NT	NT
				C	>128/16	>128/16	32/16	32/16	+	Ra
7	M/11	Y/N/N/N	N/N/retro.	A	0/0	32/0	0/0	0/0	NT	NT
				C	0/>128	16/32	0/32	0/32	+	NC
8	F/47	Y/Y/Y/Y	N/mult./groin	A	0/0	32/0	0/16	0/16	+	NC
				C	>128/16	>128/0	32/8	32/8	NT	NT
9	M/5	Y/N/N/N	Y/thumb/ axillary	C	NT/NT	NT/NT	0/0	0/0	Ra	NT
10	M/44	Y/Y/Y/N	N/shoulder/ axillary	C	32/32	>128/>128	0/0	0/0	+	Ra
11	F/10	N/N/N/N	N/N/trunk	C	0/0	0/0	0/0	0/0	+	Ra
12	M/50	N/N/N/N	N/N/N	C	0/0	0/0	0/8	0/16	+	Ra

*T, traveler; lymph., lymphadenopathy; IgG, immunoglobulin G; WB = Western blot; WB ads., WB after cross-adsorption that removed antibodies to *R. conorii*; Y, yes; N, no; A, acute phase; NT, not tested; C, convalescent phase; +, positive for *R. africae* and *R. conori*i; Ra, positive for *R. africae*; NC, not conclusive; retro., retroauricular; mult., multiple. †Ratio of titers.

Tick vectors of *R*. *africae* attack humans throughout the year. The proportion of patients having multiple eschars, which indicate the aggressive behavior of the tick, varies from 21% (*6*) to 54% (*2*). The 100% attack rate observed in this study emphasizes the risk for ATBF in sub-Saharan travelers. In our study group, only 2 persons had multiple eschars, but serologic analysis showed that all patients were infected with *R*. *africae*. Most cases of ATBF have a benign and self-limiting course with fever, headache, myalgia, and a skin rash. However, patients who are not treated show prolonged fever, reactive arthritis, and subacute neuropathy (*7*).

The long-term sequelae of ATBF remain to be established. Early treatment would not likely have prevented these complications. Jensenius et al. reported that travel from November through April was a risk factor for ATBF (*1*). The travelers in our study visited Swaziland in May. We speculate that tick bites were likely caused by larvae or nymphs, which are often unrecognized stages. Many affected travelers may not seek medical attention or may have received a wrong diagnosis. Therefore, surveillance based only on reported cases is likely to underestimate the true incidence of travel-associated *R*. *africae* infection.

## References

[1] Jensenius M, Fournier PE, Kelly P, Myrvang B, Raoult D. African tick bite fever. Lancet Infect Dis. 2003;3:557–64. 10.1016/S1473-3099(03)00739-4 12954562

[2] Raoult D, Fournier PE, Fenollar F, Jensenius M, Prioe T, de Pina JJ, *Rickettsia africae*, a tick-borne pathogen in travelers to sub-Saharan Africa. N Engl J Med. 2001;344:1504–10. 10.1056/NEJM200105173442003 11357153

[3] Consigny PH, Rolain JM, Mizzi D, Raoult D. African tick-bite fever in French travelers. Emerg Infect Dis. 2005;11:1804–6. 16422013 10.3201/eid1111.050852 PMC3367338

[4] Jelinek T, Loscher T. Clinical features and epidemiology of tick typhus in travelers. J Travel Med. 2001;8:57–9. 11285163 10.2310/7060.2001.24485

[5] Fournier PE, Roux V, Caumes E, Donzel M, Raoult D. Outbreak of *Rickettsiae africae* infections in participants of an adventure race in South Africa. Clin Infect Dis. 1998;27:316–23. 10.1086/514664 9709882

[6] Jensenius M, Fournier PE, Vene S, Hoel T, Hasle G, Henriksen AZ, African tick bite fever in travelers to rural sub-Equatorial Africa. Clin Infect Dis. 2003;36:1411–7. 10.1086/375083 12766836

[7] Jensenius M, Fournier PE, Fladby T, Hellum KB, Hagen T, Prio T, Sub-acute neuropathy in patients with African tick bite fever. Scand J Infect Dis. 2006;38:114–8. 10.1080/00365540500321579 16449002

